# High-Contrast Imaging of Cholesterol Crystals in Rabbit Arteries Ex Vivo Using LED-Based Polarization Microscopy

**DOI:** 10.3390/s18041258

**Published:** 2018-04-19

**Authors:** Seonghee Cho, Kyungmin Kim, Taehoon Kim, Hyoeun Park, Jin-Moo Kim, SeungHoon Lee, YeonSu Kang, Kiyuk Chang, Chulhong Kim

**Affiliations:** 1 School of Interdisciplinary Bioscience and Bioengineering, Pohang University of Science and Technology (POSTECH), Pohang 37673, Korea; shcho89@postech.ac.kr; 2 Department of Creative IT Engineering, Pohang University of Science and Technology (POSTECH), Pohang 37673, Korea; kyungmin10@postech.ac.kr; 3 Division of Cardiology, Department of Internal Medicine, College of Medicine, The Catholic University of Korea, Seoul 06591, Korea; viventium@naver.com (T.K.); heparkharu@gmail.com (H.P.); amyeonsohon@nate.com (J.-M.K.); solomon82831@hanmail.net (S.H.L.); dustn0898@naver.com (Y.S.K.)

**Keywords:** atherosclerosis, cholesterol crystal (Chcs), polarization microscopy

## Abstract

Detection of cholesterol crystals (Chcs) in atherosclerosis disease is important for understanding the pathophysiology of atherosclerosis. Polarization microscopy (PM) has been in use traditionally for detecting Chcs, but they have difficulty in distinguishing Chcs with other crystalline materials in tissue, such as collagens. Thus, most studies using PM have been limited to studying cell-level samples. Although various methods have been proposed to detect Chcs with high specificity, most of them have low signal-to-noise ratios, a high system construction cost, and are difficult to operate due to a complex protocol. To address these problems, we have developed a simple and inexpensive universal serial bus (USB) PM system equipped with a 5700 K cool-white light-emitting diode (LED). In this system, Chcs are shown in a light blue color while collagen is shown in a yellow color. More importantly, the contrast between Chcs and collagens is improved by a factor of 2.3 under an aqueous condition in these PM images. These imaging results are well-matched with the ones acquired with two-photon microscopy (TPM). The system can visualize the features of atherosclerosis that cannot be visualized by the conventional hematoxylin and eosin and oil-red-o staining methods. Thus, we believe that this simple USB PM system can be widely used to identify Chcs in atherosclerosis.

## 1. Introduction

Studies have shown that atherosclerosis is one of the leading causes of death from stroke and ischemic heart disease [1,2,3]. According to decades of research, cholesterol-related inflammatory responses are a major cause of atherosclerosis disease [4,5]. Therefore, many studies have focused on studying lipid-related inflammation processes to understand atherosclerosis. Lipid staining (i.e., oil-red-o, ORO), macrophage staining (i.e., RAM11), and typical cell staining (i.e., hematoxylin and eosin, H&E) have been widely used to study the pathophysiology of atherosclerosis. In addition, Finn et al. showed that a thin cap fibro-atheroma (TCFA) is a major factor for unexpected cardiac death [2]. Thus, finding the patient’s TCFA is critical for predicting vulnerable plaque and thus managing possible unexpected sudden death [6]. However, our body’s ability to heal a thin fibrous cap into a thick fibrous cap and the marks of multiple ruptures in the human coronary arteries suggest that thin fibrous caps and lipid concentrations alone cannot be a definite feature of sudden cardiac death. This means that TCFA is maybe not critical marker for sudden cardiac death [7].

Recently, cholesterol crystals (Chcs) have attracted attention as a key factor in predicting the growth of necrotic cores and plaque ruptures [8]. According to recent studies, in addition to atherosclerosis, Chcs can also cause mechanical rupture [9,10]. Thus, an imaging technique that can observe the pathophysiology of Chcs is essential for studying the atherosclerotic burden in the tissues. However, it is difficult to visualize Chcs using traditional staining methods, such as ORO and H&E, because dyes cannot directly stain crystals and Chcs are often removed during the staining process [11]. Polarizing microscopy (PM) is one of the most common methods for easy, simple, and convenient detection of Chcs [12,13,14]. However, it is difficult to distinguish Chcs from other crystalline materials, such as collagen, when Chcs are overlapped with the crystal structure. As alternatives, filipin staining, bodipy C12 staining, two-photon microscopy (TPM), and stimulated Raman spectroscopy (SRS) have been used to distinguish Chcs from other compositions [15,16]. However, these methods suffer from difficulty in operating the system due to a low signal-to-noise ratio (SNR), a high system construction cost, a narrow field of view (FOV), low specificity, and complicated protocols. Thus, there is a pressing need to develop a convenient histological protocol for observing Chcs with high sensitivity and specificity to study atherosclerosis [17].

To meet these goals, we have developed a simple and inexpensive PM system based on a simple universal serial bus (USB) microscope to reliably image Chcs and distinguish them from other crystal structures. This system uses a cool white (bluish white) light-emitting diode (LED) light of 5700 K to improve the detection sensitivity of Chcs. In this paper, we present our system and observation results using ex vivo rabbit abdominal aorta samples. We verified our imaging results with the TPM images. In addition, we compared our PM images with the images from other staining methods to show the advantages of our system. We will present our system and results in the next few sections.

## 2. Materials and Methods

### 2.1. Polarization Microscopy System

**Figure 1:**
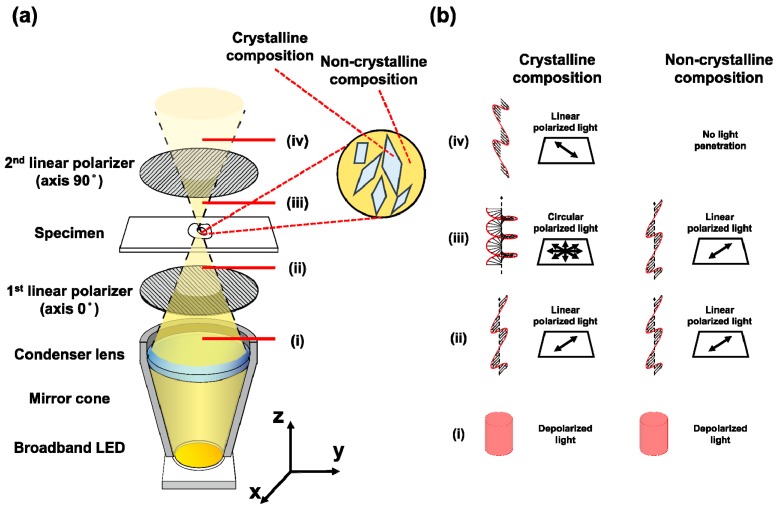
The (**a**) schematic and (**b**) principle of a polarization microscopy system. (i) space between the first polarized and condenser lens; (ii) space between the specimen and the first polarizer; (iii) space between the second polarizer and specimen; (iv) space between the aperture of the microscope and the second polarizer. LED = light-emitting diode.

The schematic of a PM system is shown in Figure 1a. We used a three-axis mechanical stage (Line Tool Co. A-LH 3-axis linear conversion stage, Allentown, PA, USA) as a manual microscope framework. The main frame for the specimen and polarizers were custom-made using a three-dimensional (3D) printer. Two USB-typed digital microscopes with low (Anmo electronics, AM4013MZTL, $629, Hsinchu, Taiwan) and high (Anmo electronics, AM4515T8, $849, Hsinchu, Taiwan) magnifications were used as the main PM systems. A linear polarizer film was used as the polarizer and analyzer. Since halogen light sources have difficulty covering wavelengths below 500 nm, we used a 5700 K white high-power LED (Lumileds, L2C2-57701208E1500, $25, Amsterdam, Netherlands) as a light source. This LED covers the full spectrum of visible light range from 400 to 700 nm. Previous studies have shown that the absorption coefficient of collagen is higher than Chcs in visible light, especially at wavelengths below 500 nm [18,19]. Thus, the LED used in our system with the spectrum range of 400 to 700 nm will be able to distinguish Chcs from collagens because of differences in the absorption coefficient between them.

As shown in Figure 1a,b, only the light passing through the crystalline composition can be detected using a microscope. Initially, the light emitted from the LED is linearly polarized by the first polarizing film. The birefringence of crystallized materials (i.e., Chcs and collagens) causes phase retardation in incident light resulting in circularly polarized light. The circularly polarized light has a vertical component of linearly polarized light and passes through the second polarizing film. The light transmitting through the second polarizing film is detected by a digital microscope to form images of crystallized substances, such as collagen and Chcs. The main advantages of our system are: (1) the cost of our system is relatively low because of cost-effective USB microscopes and LEDs. (2) No labeling is required, unlike in bodipy and filipin staining methods. (3) The imaging protocol is relatively simple and easy to use compared to TPM or fluorescence staining methods since no dark environment is required. (4) The specificity for detecting Chcs is high since the system can highlight Chcs with strong contrast. (5) Thanks to the wide FOV and high SNR of our system, real-time imaging is possible and does not require signal averaging.

### 2.2. Ex Vivo Sample Preparation

Eight male rabbits weighing 3–4 kg were used in the atherosclerosis model. All rabbits were fed a 1% cholesterol diet (Dyets Inc., Bethlehem, PA, USA) for 2 weeks. Subsequently, surgical procedures were performed to create an arterial plaque. Rabbits were anesthetized with Zoletil and Rompun (300 uL:200 uL/kg). A Hi-torque balanced middleweight guidewire (Abbott, Chicago, IL, USA) was inserted through the right carotid artery into the common carotid artery and the brachial artery. A 3F Fogarty balloon catheter introduced through the common carotid artery (Edwards Lifesciences, Irvine, CA, USA) was used to damage the arterial wall. The inflated balloon retracted and caused injury. This procedure was repeated five times on the abdominal aortic wall. After surgery, all rabbits received a 1% cholesterol diet for 3 to 5 weeks. For imaging experiments, atherosclerotic rabbits were sacrificed and the arteries were extracted, fixed with paraformaldehyde (4% in phosphate-buffered saline), and sectioned.

### 2.3. Ethical Statement

The study was conducted in accordance with the Declaration of Helsinki, and the protocol was approved by the Ethics Committee of The Catholic University of Korea (IACUC 2016-0138-01).

### 2.4. Experimental Procedures

Five-μm-thick sections were prepared and made into three groups: an ORO-stained group, an H&E-stained group, and an unstained group. Each group consisted of seven samples. Both ORO- and H&E-stained samples were imaged using a conventional histology microscopy system (Leica, SCN400, Wetzlar, Germany). The unstained samples were imaged using our developed PM system and the results were validated with the images acquired with a TPM system (Leica, TCS SP5, Wetzlar, Germany). Unstained samples were imaged under aqueous and non-aqueous conditions in PM imaging. A water droplet was added to the sample and covered with glass to create an aqueous condition. A wavelength of 900 nm was used as a TPM excitation source. Due to the limited SNR of TPM, four lines were averaged and four frames were accumulated during image acquisition. Because the FOV was limited, 10–15 TPM images were stitched to provide a full arterial cross-sectional image. All PM images are normalized to the red, green, and blue pixel values of the background to compensate for the spectral differences of the LED source and complementary metal–oxide–semiconductor (CMOS) detector.

## 3. Results

### 3.1. Cholesterol Crystal Detection

**Figure 2:**
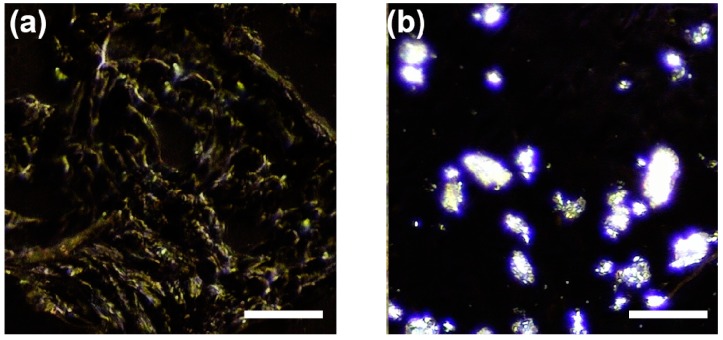
Polarization microscopy (PM) images of controlled samples. (**a**) collagen tissue from pig skin and (**b**) cholesterol crystal. Scale bars: 50 μm.

Due to the difference in optical absorption, our system displays Chcs in a cool white color and normal aortic tissues (mainly collagen) in a dark yellow color [18,19]. Figure 2 shows this characteristic from the controlled samples. Figure 2a is the PM image obtained from pig skin tissue composed mainly of collagen fibers. Figure 2b is a PM image obtained from cholesterol crystals. To prepare the sample slides, the cholesterol powder (Sigma Aldrich, C8667, St. Louis, MO, USA) was fixed in a gelatin gel and cut to a thickness of 5 μm.

**Figure 3:**
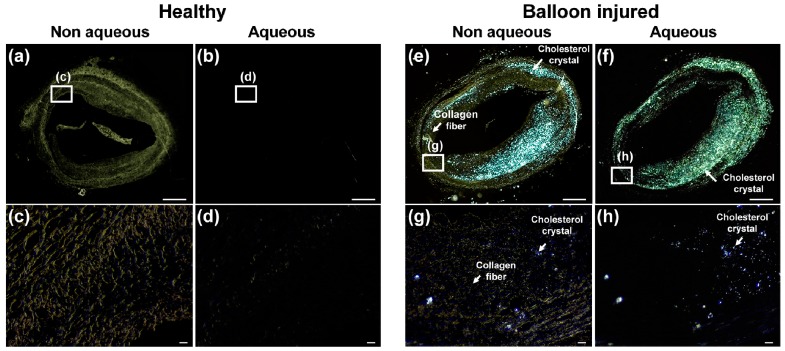
Polarization microscopy (PM) images of a sectioned rabbit artery sample. (**a**–**d**) are PM images of a healthy site of the artery and (**e**–**h**) is a balloon-injured site of the artery. Images were acquired under a non-aqueous (**a**,**f**) and an aqueous condition (**b**,**g**). (**c**,**d**) are highly magnified images of white-boxes in (**a**,**b**), respectively. (**g**,**h**) are highly magnified images of white-boxes in (**e**,**f**), respectively. Scale bars: (**a**,**b**,**e**,**f**) 500 μm and (**c**,**d**,**g**,**h**) 20 μm.

We can observe the same characteristics from a rabbit artery sample. We acquired PM images from a healthy site of an artery and from the balloon-injured site. This result is shown in Figure 3. In Figure 3, we show the typical characteristics of Chcs and collagen of a rabbit artery sample. Figure 3a–d are PM images achieved from a healthy site and (e) to (h) are PM images achieved from the balloon-injured site. In Figure 3a, the dark-yellow components are collagen tissues. In the aqueous condition (Figure 3b), the collagen components are no longer visible. In Figure 3c,d, magnified images show more detail change in the image. In Figure 3d, the dark-yellow components are collagen tissues and the cool-white components are Chcs. In the aqueous condition (Figure 3f), the collagen components are no longer visible and Chcs are highlighted. This phenomenon is clearly visible in the high magnification images shown in Figure 3g,h. The Chcs signals in the aqueous solution are enhanced as shown in Figure 3h while suppressing the collagen signal (yellow), providing a very good contrast between them. When the collagen absorbs H_2_O molecules, the refractive index of collagen was changed from 1.547 to 1.375. The refractive index at high humidity is similar to the refractive index of water at 1.333 [20,21]. Based upon this property, the collagen signal is suppressed and the Chcs signal is highlighted under the aqueous condition. The effect of the water is well-visualized in Video S1 provided in the supplementary document.

**Figure 4:**
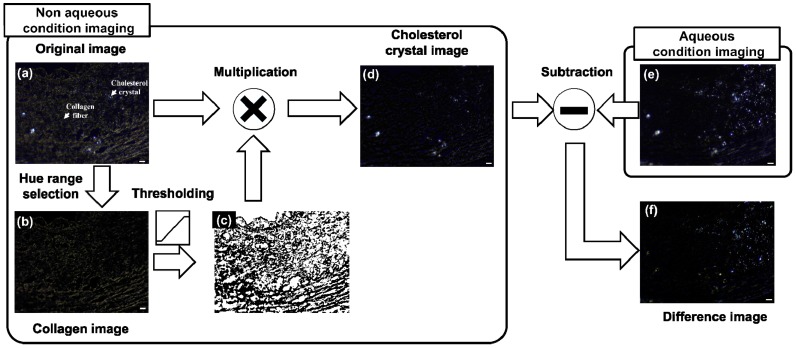
Schematic describing process selecting cholesterol crystals (Chcs) from a non-aqueous condition image and visualizing the difference between the non-aqueous condition image and an aqueous condition image by making a difference image. (a) Original image of non-aqueous condition; (**b**) Collagen image extracted from (**a**); (**c**) Collagen removal mask; (**d**) Cholesterol crystal image; (**e**) Original image from aqueous condition; and (**f**) difference image which is generated by subtracting (**d**) and (**e**). Scale bars: 20 μm.

To compare aqueous and non-aqueous conditions quantitatively, we compared the area of Chcs regions under both conditions in PM images. The details of quantitative comparison are shown in Figure 4. Initially, the Chcs regions were selected from PM images acquired under both aqueous and non-aqueous conditions. In the PM image obtained from the non-aqueous condition, the collagen signal was removed based on the hue information to enhance the Chcs signal. The process for enhancing the Chcs signal was as follows: (1) a collagen area was selected based on hue information (Figure 4b), (2) a collagen removal mask was generated (Figure 4c) and multiplied with the original image, and (3) the Chcs region was segmented. No additional image processing was needed on the PM image obtained under the aqueous condition. To visualize the difference between these two images, the Chcs region from the non-aqueous condition was subtracted from the PM image obtained under the aqueous condition. The measured areas of the Chcs regions for the non-aqueous and the aqueous condition were 2147 μm^2^ and 6024 μm^2^, respectively. This result shows that the study of a sample in aqueous solution is a key step to improve Chcs contrast in the PM image.

We quantify the PM image contrasts using both aqueous and non-aqueous conditions as follows:

C = (S_Chcs_ − S_B_)/S_B_ (1)

where C is the image contrast, SChcs is the signal from Chcs, and S_B_ is the signal value of the adjacent pixels. To measure the overall contrast in an image, we calculated the intensity of each pixel. All 8-bit R, G, and B pixels were summed in a one-dimensional matrix to form a gray-shaded image of 768 levels. The intensities of collagen, Chcs, and background were 79 ± 37, 403 ± 132, and 38 ± 4, respectively. The average contrast of Chcs to collagen was 4.1 (Figure 4a) and that of Chcs to background was 9.6 (Figure 4e). This result shows that by adding water to the sample, the PM image contrast can be improved by a factor of 2.3.

**Figure 5:**
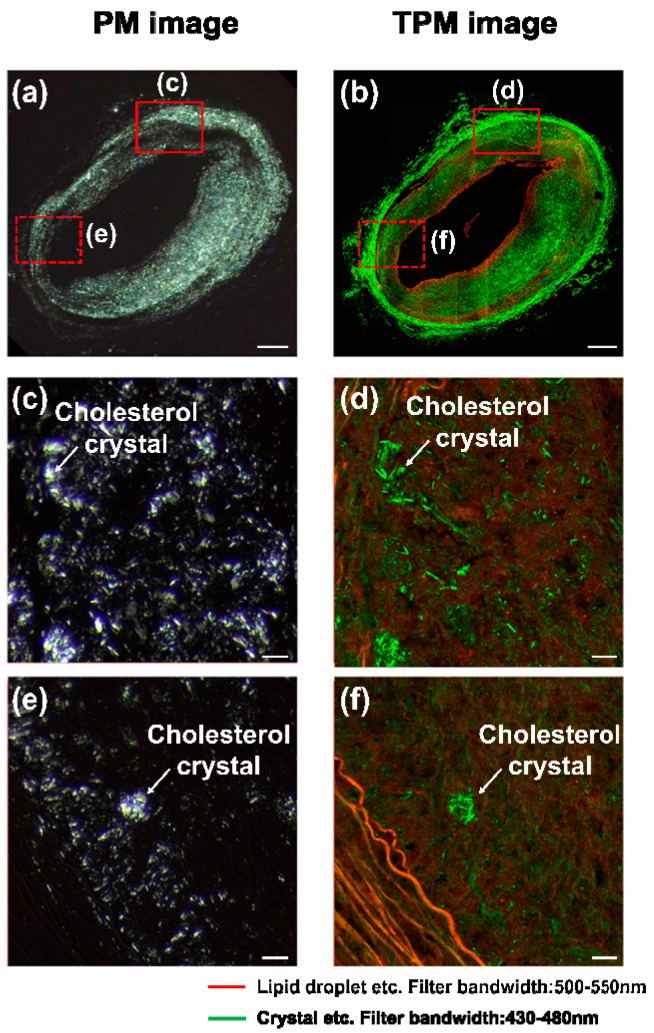
Polarization microscope (PM) image (left column) and two-photon microscope (TPM) image (right column). A sectioned rabbit artery sample imaged by using (**a**) PM and (**b**) TPM. Highly magnified images of the sample by using (**c**,**e**) PM and (**d**,**f**) TPM. Scale bars: (**a**,**b**) 500 μm and (**c**–**f**) 20 μm.

To validate our PM imaging results, we imaged the same sample using TPM. The PM image of the artery sample is shown in Figure 5a and the TPM image of the same artery sample is shown in Figure 5b. In the TPM images, the second harmonic signals generated by Chcs and collagen are displayed in green and the fluorescent signals are displayed in red. In Figure 5b, the adventitia layer composed of a high-density collagen network exhibited strong second harmonic signals. The intima layer showed a fluorescence signal and a relatively lower second harmonic signal than that of the adventitia layer. The intensity of the second harmonic signal in the intima layer showed a good correlation with Chcs in the PM image. This correlation is more evident in the magnified images shown in Figure 5c,d of the region marked with a solid red box of (a) and (b), respectively. In the magnified image, green needle- or diamond-shaped structures are Chcs. As shown in Figure 5c,d, Chcs are visible in the PM image with minimal background (c) while the Chcs signals in the TPM image are blended with other second harmonic signals generated from the collagen and other fluorescent signals. In the further magnified image, shown in Figure 5e,f, we can see this trend even more clearly. A small Chcs band is visible in the left bottom of Figure 5e, but the visibility is decreased in (f) due to the fluorescence signals from other proteins. Based on this observation, we can conclude that our PM image results are better in visualizing Chcs than those of the TPM image results.

### 3.2. Comparison with Conventional Histological Methods

**Figure 6:**
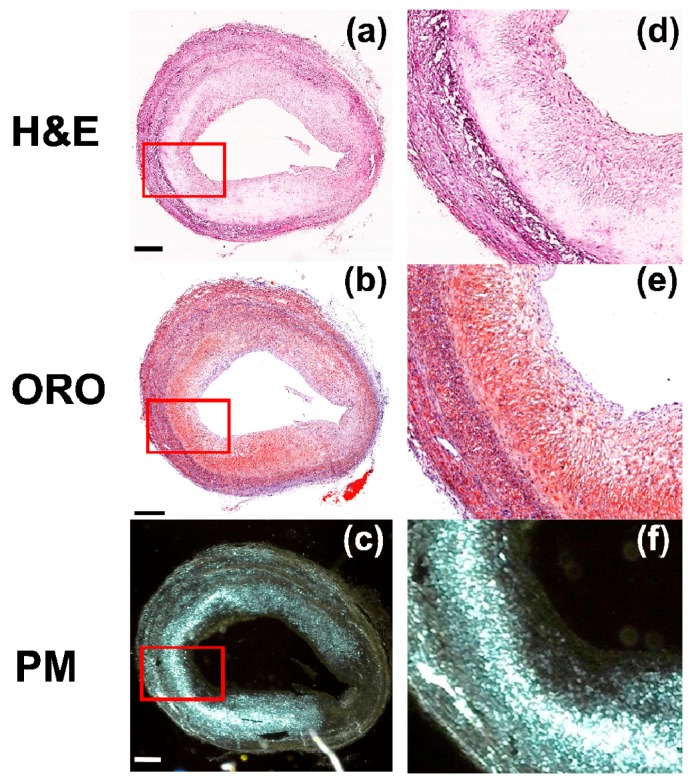
Microscopic images of artery samples with advanced fibroatheroma stained with (**a**) Hematoxylin and Eosin (H&E) and (**b**) Oil-Red-O (ORO). (**c**) Polarization microscopic image of an unstained sample. (**d**–**f**) are magnified images of the red box in (**a**–**c**), respectively. Scale bars: (**a**–**c**) 500 μm and (**d**–**f**) 200 μm.

We compared triple histological imaging methods, such as H&E, ORO, and PM, to distinguish cell viability, lipid, and Chcs distribution. This common tendency was well-observed in severe atherosclerosis samples of the artery. Figure 6 is a set of histological images of an arterial sample at a severe stage of atherosclerosis. An image stained with H&E is shown in Figure 6a, an image stained with ORO in Figure 6b, and an unstained sample acquired by PM in Figure 6c. In all of these images, fatal necrosis was observed and the necrotic region was highly correlated with the distribution of Chcs and lipid. The magnified images of the solid red boxes in (a), (b), and (c) are shown in Figure 6d–f, respectively. The strong correlation between the necrosis, Chcs, and lipid is even more obvious in these magnified images.

**Figure 7:**
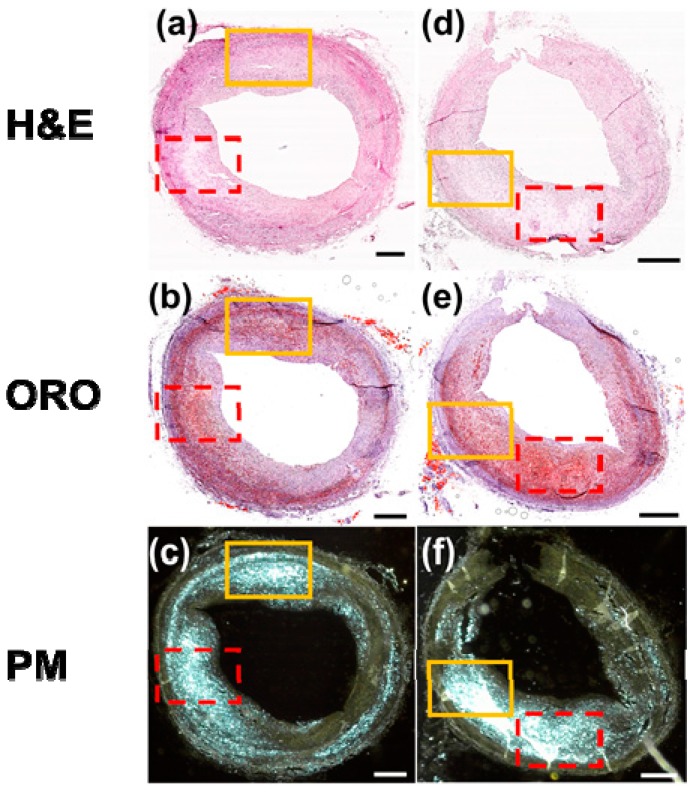
Microscopic images of artery sample with a relatively early stage of atheroma stained with (**a**,**d**) H&E and (**b**,**e**) ORO and (**c**,**f**) PM images of unstained samples. All scale bars: 500 μm.

These staining methods were successful for a severe stage of atherosclerosis. However, this will not always be true for all stages of atherosclerosis. To illustrate this, a histological image set of arterial samples at a relatively early stage of atherosclerosis for various staining methods is shown in Figure 7. Figure 7a,d show H&E-stained images, (b) and (e) show ORO-stained images, and (c) and (f) show PM images of unstained samples. In Figure 7a–c, the Chcs distribution in the red-dotted boxes matches well with the necrotic core and the high lipid concentration region. In Figure 7d–f, the lipid concentration is highest in the red dot box and this area matches well with the Chcs distribution and the necrotic area. These results for red boxes are similar to the Figure 6 results. However, in the case of the solid yellow boxes, it showed a result different from the red dot boxes. In the solid yellow boxes of Figure 7a–c, negligible necrotic areas and mild lipid concentrations were observed in areas with a high concentration of Chcs. In the solid yellow boxes of Figure 7d–f, Chcs signals were much higher than those in the red dot box area with a lower lipid concentration and higher cell viability.

## 4. Discussion

While it is impossible to obtain a sample from living human arteries, it is still important to detect Chcs in human tissues. In terms of research, histological studies of cadavers are useful to researchers in understanding atherosclerosis and related pathology [2,7,22]. Histological examination of cadaver samples is also important for the development of new tools for the diagnosis of atherosclerosis [23,24,25,26]. In clinical aspects, Chcs embolization is one of the most important diseases associated with Chcs. A biopsy of the kidney or other tissue is used to find Chcs in the tissue to diagnose Chcs emboli [27].

Staining methods are simple and have advantages for visualizing physiological information of tissues, tissue structures, protein distribution, and lipid distribution amongst other things. However, they have limitations, as described in the previous paragraph, for accurately detecting Chcs at various levels of atherosclerosis. Using our simple PM system, however, we could successfully detect Chcs in the atherosclerotic samples. Thus, our tool is potentially useful for atherosclerosis management by visualizing Chcs directly, which is a unique characteristic of atherosclerotic plaque.

## 5. Conclusions

In order to achieve a better understanding of the pathological processes involved in atherosclerosis due to cholesterol crystals (Chrs), it is necessary to image and observe Chcs directly. In this study, we presented a simple PM system to image Chrs with high contrast and also to differentiate them from collagen. We also performed a simple feasibility study on a rabbit’s artery to confirm the system’s capability. Based on our arterial tissue studies results, we found that our system is well-suited to analyze Chcs at the tissue level. Our system is also easy to use and cost-effective compared to other developed methods. In addition, since Chcs is associated with some cholesterol metabolism-related diseases as well as atherosclerosis, the simple microscopy method introduced in this study could help to study cholesterol crystals on a tissue scale. As a next step, we will expand our study to include human in vitro samples to see how our system performs with a real-world clinical problem.

## Supplementary Materials

The following are available online at http://www.mdpi.com/1424-8220/18/4/1258/s1, Video S1: Effect of water on polarization microscopy.
